# Peripartum Cardiomyopathy in India

**DOI:** 10.7759/cureus.105892

**Published:** 2026-03-26

**Authors:** Soumya S Patil, Vidya Jadhav, Chidanand Chivate, Rahul Mahadik

**Affiliations:** 1 Obstetrics and Gynaecology, Bharati Vidyapeeth (Deemed to Be University) Medical College and Hospital, Sangli, IND; 2 Internal Medicine, Bharati Vidyapeeth (Deemed to Be University) Medical College and Hospital, Sangli, IND; 3 Cardiology, Bharati Vidyapeeth (Deemed to Be University) Medical College and Hospital, Sangli, IND

**Keywords:** cardiogenic shock, dilated cardiomyopathy (dcm), high-risk pregnancy, left ventricular ejection fraction, maternal mortality, medical termination of pregnancy, peripartum cardiomyopathy, peripartum period, preeclampsia (pe), systolic heart failure

## Abstract

Background

Peripartum cardiomyopathy (PPCM) is characterized by left ventricular systolic dysfunction occurring toward the end of pregnancy or in the months following delivery. In India, delayed recognition due to overlap with physiological pregnancy symptoms often leads to advanced disease at presentation. This study aimed to evaluate the clinical profile, echocardiographic findings, management, and maternal-neonatal outcomes of PPCM cases managed at a tertiary care center.

Methodology

This retrospective observational study was conducted over three years in the Department of Obstetrics and Gynaecology at a tertiary care center. A total of 11 women fulfilling the European Society of Cardiology diagnostic criteria for PPCM were included. Demographic data, risk factors, clinical presentation, laboratory parameters, echocardiographic findings, management details, and maternal and neonatal outcomes were analyzed using descriptive statistics.

Results

The mean maternal age was 27.4 ± 6.1 years. Most patients were unbooked referrals (90.9%), and 54.5% presented in the antepartum period. Breathlessness was the predominant symptom (90.9%), with 90.9% presenting in New York Heart Association class III-IV. Preeclampsia was the most common associated risk factor (54.5%). Mean left ventricular ejection fraction (LVEF) at diagnosis was 35.3% (range = 15-45%). Severe left ventricular dysfunction (<30%) was observed in half of the cases. Intensive care unit admission was required in the majority, with mechanical ventilation in 27.3% and cardiogenic shock in 33.3%. Maternal mortality was 9.1%. At six months, 80% of survivors demonstrated recovery of left ventricular function (LVEF >50%), while 20% had persistent dysfunction. Preterm birth occurred in 44.4% of deliveries. Neonatal mortality was 11.1%, and neonatal intensive care unit admission was required in 22.2%.

Conclusions

PPCM remains an under-recognized yet potentially life-threatening cause of heart failure in pregnancy. Most patients present with severe left ventricular dysfunction but demonstrate significant recovery with timely multidisciplinary management. Increased clinical awareness, early diagnosis, and structured follow-up are essential to improve maternal and neonatal outcomes.

## Introduction

Peripartum cardiomyopathy (PPCM) is a form of systolic heart failure due to cardiomyopathy [1]. PPCM was earlier described as the onset of symptomatic heart failure during the last month of pregnancy or within five months after childbirth [2,3]. Later observations suggested that women who developed similar clinical features earlier in gestation could not be differentiated from those meeting the classical criteria. In view of these findings, the Heart Failure Association of the European Society of Cardiology (ESC) Working Group updated the definition in 2010, characterizing PPCM as an idiopathic cardiomyopathy presenting with heart failure due to left ventricular systolic dysfunction occurring toward the late stages of pregnancy or during the postpartum period, after exclusion of other identifiable causes of heart failure [3].

ESC guidelines in 2025 defined PPCM as “heart failure with reduced left ventricular ejection fraction (LVEF) <45%, without any other cause of HF, that occurs mainly during the peripartum period or in the months following delivery, termination, or miscarriage. It is a diagnosis of exclusion and requires urgent management.” The global incidence of PPCM has been reported to be 1 in 2,000 [4]. The incidence of PPCM in the Indian population has been reported to be 1 in 1,340 live births. Indian women develop PPCM at a mean age of 27 years and most commonly in the postpartum period (60% cases) [5].

In India, the true burden of PPCM is under-recognized due to the overlap of its symptoms with normal physiological changes of pregnancy and the postpartum period, a low index of clinical suspicion, and delayed referrals from peripheral healthcare centers. Common symptoms such as breathlessness, pedal edema, and fatigue are often misattributed to anemia, hypertensive disorders of pregnancy, or postoperative changes following cesarean delivery, resulting in delayed diagnosis and advanced disease at presentation [2]. The incidence of PPCM is increasing due to improvement in diagnosis, along with an increase in maternal age, preeclampsia, multiparity, and multiple gestations [6].

PPCM places a disproportionate burden on tertiary care obstetric services, as affected women often require intensive care unit (ICU) admission, advanced cardiac support, and multidisciplinary management. Most patients are referred in a critically ill state, prolonging ICU stays, and the need for advanced investigations and therapies further contributes to increased healthcare costs, imposing a significant financial burden on the affected families. Therefore, in this study, we aim to report and evaluate the cases of PPCM and its clinical course.

## Materials and methods

This was a single-institution, retrospective, observational study conducted in the Department of Obstetrics and Gynaecology at Bharati Vidyapeeth (Deemed to Be University) Medical College and Hospital, Sangli, Maharashtra, India. The study included records of patients diagnosed with PPCM, including those with a history of PPCM, recurrence, late postpartum presentation, and outcomes following recovery, over a three-year period. The Institutional Ethics Committee waived the requirement for informed consent. Patient confidentiality was strictly maintained, and all identifiable information was anonymized before analysis.

Study population

Women fulfilling the ESC diagnostic criteria for PPCM were included. PPCM is a diagnosis of exclusion. In this study, PPCM was defined as heart failure with reduced LVEF <45%, without any other cause of heart failure, that occurs mainly during the peripartum period or in the months following delivery, termination, or miscarriage. Patients with known pre-existing cardiomyopathy, congenital or valvular heart disease, ischemic heart disease, pulmonary embolism, or other identifiable causes of heart failure were excluded.

Data collection

Clinical, laboratory, and imaging data were extracted from hospital records using a predefined proforma. Baseline demographic characteristics, including age, parity, gestational age at delivery, mode of delivery, referral status, and obstetric risk factors such as hypertensive disorders of pregnancy, anemia, and multiple gestations, were documented. Clinical presentation details included timing of symptom onset in relation to pregnancy or the postpartum period, presenting symptoms (breathlessness, orthopnea, pedal edema), and the New York Heart Association (NYHA) functional class at presentation. Vital parameters and requirements for ICU admission and ventilatory support were noted. Laboratory parameters included complete blood count, renal and liver function tests, inflammatory markers (C-reactive protein (CRP)), cardiac biomarkers (N-terminal pro-B-type natriuretic peptide (NT-proBNP), troponin-I, creatine kinase-myocardial band (CK-MB)), whenever performed, to rule out etiologies of heart failure.

Records of baseline two-dimensional transthoracic echocardiography at the time of diagnosis, discharge, and during subsequent follow-up to assess recovery or persistence of left ventricular dysfunction were collected. Changes in LVEF over time were documented to evaluate myocardial recovery. Echocardiographic parameters included LVEF, global hypokinesia, mitral and tricuspid regurgitation, pulmonary artery systolic pressure, dilatation of chambers, pericardial effusion, and presence of intracardiac thrombus.

Statistical analysis

Descriptive statistics were used to summarize demographic, clinical, echocardiographic, and outcome variables. Continuous variables were expressed as mean ± standard deviation or median with range, and categorical variables as frequencies and percentages.

## Results

This study included 11 patients with PPCM presenting across varying gestational ages. The mean maternal age was 27.4 ± 6.1 years (range = 19-36 years), with 10/11 (90.9%) cases presenting as unbooked, including four referred after a lower-segment cesarean section performed outside, while only one patient was booked. The majority of patients (n = 6/11, 54.5%) presented in the antepartum period. Most deliveries were by cesarean section, and two by the vaginal route. ICU admission was required in the majority, reflecting severe disease at presentation. Clinical details of the patients are summarized in Table 1. Baseline characteristics are presented in Table 2.

**Table 1:** Clinical details of patients. LSCS = lower-segment cesarean section; LAMA = left against medical advice; CRP = C-reactive protein; NYHA = New York Heart Association; MR = mitral regurgitation; TR = tricuspid regurgitation; NT-proBNP = N-terminal pro-B-type natriuretic peptide; CK-MB = creatinine kinase myocardial band; HDP = hypertensive disorders of pregnancy; IUGR = intrauterine growth restriction; HTN = hypertension

Case number	Age (years)	Symptoms	Gestational age at diagnosis	LVEF at diagnosis (%)	Investigations	Complications	Delivery and neonatal outcome	Maternal status
1	26	Developed breathlessness after two hours of LSCS	Postoperative day 0	25%	NT-proBNP = 15,300 pg/mL	Severe pulmonary artery hypertension, all chambers dilated, severe TR	Emergency LSCS at 39 weeks (contracted pelvis)	Maternal mortality within 24 hours of admission
2	19	Breathlessness	Postoperative day 2	15%	NT-proBNP = 29,200 pg/mL; troponin-I = 1.7 ng/m:; CRP >320 mg/dL	Twins	LSCS at 37 weeks (twins)	LAMA after 6 days
3	21	Breathlessness for 2 days	Postoperative day 2	40%	Limited labs available	NYHA III, mild MR and TR	LSCS at 38 weeks (non-progress of labor)	Discharged after 4 days
4	31	No complaints (history of PPCM)	Past history	Normal	All labs normal	None (history only)	LSCS at 38 weeks (non-progress of labor)	Discharged after 7 days
5	28	Breathlessness, chest pain	36 weeks	45%	NT-pro BNP = 1,513 pg/mL; troponin-I- = negative CK-MB = 32 U/L	Hypertensive disorder of pregnancy, severe MR, moderate TR, pulmonary artery hypertension	LSCS at 36 weeks for PPCM	Discharged after 10 days
6	28	Breathlessness	30 weeks	35%	NT-proBNP = >35,000 pg/mL; troponin-I = negative; CK-MB = 213 U/L	Preeclampsia, intrauterine death	LSCS at 30 weeks for PPCM	Discharged after 10 days; intrauterine death
7	22	Breathlessness	30 weeks	45%	NA	HDP, IUGR, oligohydramnios, Doppler changes	LSCS at 30 weeks	Discharged after 15 days; maternal survival; preterm neonate
8	34	Breathlessness, cough, vomiting	36 weeks	45%	CRP = 22.7 mg/dL	Multiparous, chronic HTN, oligohydramnios, NYHA III	Preterm vaginal delivery at 36 weeks	Discharged after 8 days; maternal survival
9	36	Breathlessness for 12 hours	37 weeks	20%	NT-proBNP = 20,906 pg/mL; CK-MB = 55 U/L; troponin-I = 0.3 ng/mL	HDP, all chambers dilated, mild MR and TR, PA HTN (55 mmHg)	LSCS at 37 weeks for PPCM	Discharged after 8 days; LVEF improved to 35% at 6 months; maternal survival; the baby died due to 29
10	29	Breathlessness for 12 hours	21 weeks	32%	Limited labs available	Recurrent PPCM	Medical termination of pregnancy; hysterotomy performed	Medical termination of pregnancy
11	35	Breathlessness for 2 days	Postnatal day 40	30%	Troponin-I = negative; CK-MB = 44 U/L	Twin pregnancy	LSCS at 37 weeks d/t twin pregnancy	Discharged after 5 days; maternal survival

**Table 2:** Baseline characteristics.

Characteristic	Value, n (%)
Mean maternal age (years)	27.4 ± 6.1 years (range = 19–36 years)
Age >30 years	4 (36.4%)
Unbooked referrals	10 (90.9%)
Multiparous	5 (55.6%)
Multiple gestation	2 (22.2%)
Hypertensive disorders of pregnancy	6 (54.5%)
Timing of presentation	
Antepartum	6 (54.5%)
Postpartum (postoperative day 0–2)	3 (27.3%)
Mean gestational age at delivery (weeks)	34.55 weeks
Mode of delivery	
Cesarean section	8 (72.7%)
Vaginal delivery	2 (18.2%)

Clinical symptoms

Breathlessness was the predominant presenting symptom (n = 10/11, 90.9%), presenting as dyspnea on exertion, dyspnea at rest, or orthopnea. Associated symptoms included chest pain (n = 2), palpitations, pedal edema, fever, and syncope. Preeclampsia was the most frequently associated risk factor, and two patients had multiple gestations. Most of the patients presented in NYHA Class III-IV, with SpO₂ ranging from 75% to 93% on room air at admission (Table 3).

**Table 3:** Clinical presentation and functional status. NYHA = New York Heart Association; ICU = intensive care unit

Parameter	Value, n (%)
Breathlessness	10 (90.9%)
Chest pain	2 (18.2%)
Palpitations/syncope	2 (18.2%)
Pedal edema	6 (54.5%)
NYHA Class III–IV at presentation	10 (90.9%)
SpO₂ at admission (room air)	75–93%
ICU admission required	Majority
Mechanical ventilation	3 (27.3%)
Cardiogenic shock	3 (33.3%)

Preterm births were noted in 4/11 (40%) cases, with the mean gestational age at delivery being 34.55 weeks.

Laboratory and echocardiographic findings

Laboratory investigations revealed markedly elevated cardiac biomarkers in symptomatic cases: NT-proBNP ranged from 1,513 to >35,000 pg/mL, with approximately half of the patients showing values >1,000 pg/mL. Other cardiac injury markers, including troponin-I and CK-MB, were elevated in a subset of patients in whom these investigations were performed, while inflammatory markers such as CRP were frequently raised (Table 4).

**Table 4:** Cardiac biomarkers and laboratory findings. Elevated values were defined as NT-proBNP >1,000 pg/mL, troponin-I >0.04 ng/mL, CK-MB >5 ng/mL, and CRP <0.3 mg/dL. NT-proBNP = N-terminal pro-B-type natriuretic peptide; CK-MB = creatinine kinase myocardial band; CRP = C-reactive protein

Parameter	n/N	Elevated, n (%)
NT-proBNP >1,000 pg/mL	5/11	5 (45.5%)
Troponin-I	5/11	2 (40%)
CK-MB	4/11	4 (100%)
CRP	4/11	4 (100%)

**Figure 1:**
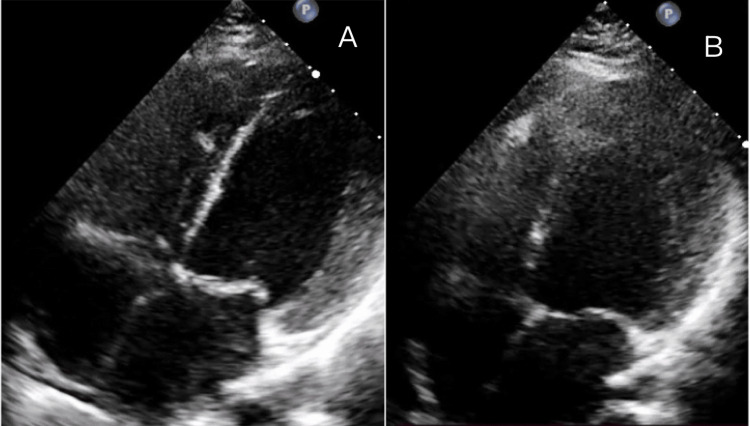
Apical four-chamber view (A) at diagnosis suggesting dilated left ventricular cavity. LVEF was diagnosed using the modified Simpson’s method. (B) At the one-month follow-up. LVEF = left ventricular ejection fraction

**Figure 2:**
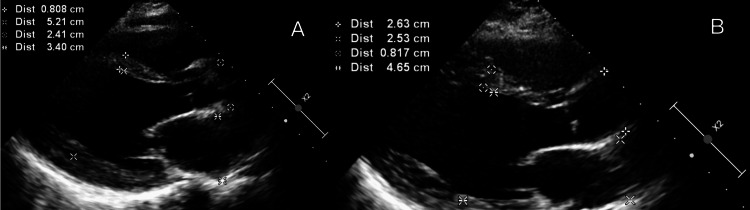
Parasternal long-axis view. (A) At diagnosis measuring left ventricular dimensions. (B) Improved left ventricular dimensions at the one-month follow-up.

**Figure 3:**
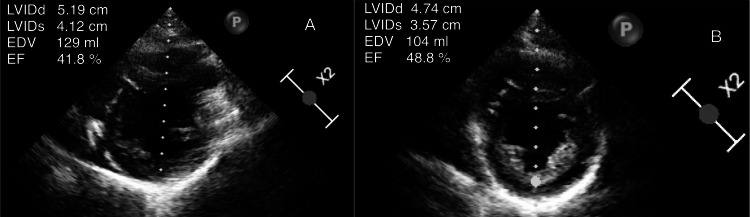
Parasternal short-axis view. (A) At diagnosis suggesting LVEF of 41.8%. (B) At the one-month follow-up with an improved LVEF of 48.8%. LVEF = left ventricular ejection fraction

Echocardiographic assessment revealed significantly reduced LVEF, ranging from 15% to 45% at diagnosis (mean = 35.3%), with severe reduction (<30%) in half of the cases. Global hypokinesia was present in all patients, accompanied by left ventricular dilatation in 4/9 (44.4%) cases. Most patients had functional, mitral, and tricuspid regurgitation (Table 5). Serial two-dimensional transthoracic echocardiography demonstrated left ventricular dilatation with reduced ejection fraction at diagnosis and subsequent improvement at the one-month follow-up (Figures 1-3).

**Table 5:** Trends and early outcomes of LVEF. LVEF = left ventricular ejection fraction; ICU = intensive care unit

Parameter	Value
Mean LVEF at diagnosis (%)	35.3 ± 9.2% (15–45%)
Mean LVEF at discharge (%)	41.4 ± 7.5% (25–45%)
Patients showing LVEF improvement at discharge	4 patients
Mean of Long-term LVEF follow-up at 6 months	52.6 ± 8.7% (35–60%)
ICU stay (days)	Variable
Hospital stay (days)	4–15

Management

**Figure 4:**
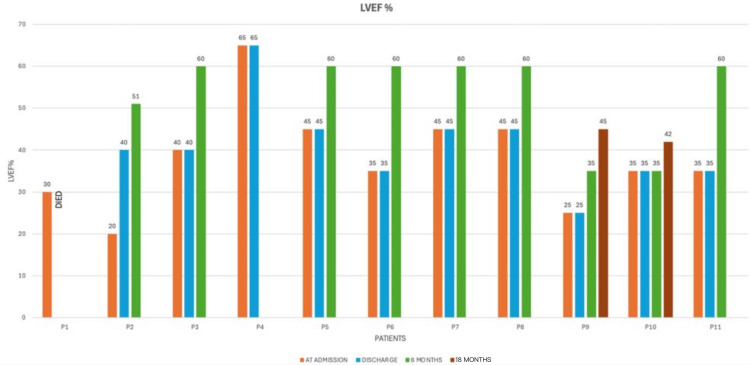
Serial changes in LVEF at admission, discharge, and six-month follow-up. Most patients demonstrated progressive improvement in LVEF over time. Eighteen-month follow-up for two patients (P9 and P10) showed partial recovery with persistent left ventricular dysfunction. LVEF = left ventricular ejection fraction

Medical management included diuretics in 10 patients and beta-blockers in seven patients each, angiotensin-converting enzyme inhibitors/angiotensin receptor blockers/angiotensin receptor-neprilysin inhibitors in four patients, mineralocorticoid receptor antagonists in eight patients, and inotropic support such as dopamine/milrinone in two cases. Three patients required mechanical ventilation, and two received anticoagulation. LVEF improved at discharge in four evaluable patients (mean = 41.4%, range = 25-45%), indicating a favorable early response to medical therapy. Long-term follow-up LVEF improved in seven patients (mean = 52.6%, range = 35-60%) at six months, showing continued improvement. LVEF was 35% in two patients at six months, showing residual disease. On long-term follow-up at 18 months, LVEF in these two patients was 45% and 42%, suggesting partial recovery (Figure 4).

Neonatal outcomes were documented in nine deliveries; one case was excluded as it represented a history of PPCM in a previous pregnancy, with the current pregnancy being uncomplicated. The second case was a medical termination of pregnancy due to recurrent PPCM with relapse in the second trimester, presenting with severe left ventricular systolic dysfunction (ejection fraction = 25%) corresponding to WHO class IV maternal cardiac risk. One case resulted in intrauterine fetal demise following maternal PPCM complicated by preeclampsia. Preterm births occurred in four (44.4%) neonates. Neonatal intensive care unit (NICU) admission was required in two (22.2%) neonates. Neonatal mortality was documented in one (12.5%) case, occurring in a neonate delivered at 37 weeks’ gestation (birth weight = 2.8 kg). The remaining seven neonates (70%) survived without documented complications, demonstrating acceptable neonatal outcomes despite the significant maternal cardiac compromise (Table 6).

**Table 6:** Maternal and neonatal outcomes MTP = medical termination of pregnancy; NICU = neonatal intensive care unit

Outcome	Value, n (%)
Maternal survival	10/11 (90.9%)
Maternal mortality	1/11 (9.1%)
Total deliveries analyzed	9
Live births	7 (77.8%)
Neonatal death	1 (11.1%)
Intrauterine death	1 (11.1%)
MTP	1
NICU admission	2 (22.2%)
Mean birth weight (kg)	2 (0.75–3.0)
Preterm birth	4 (44.4%)

Outcomes

Maternal mortality occurred in one case, while 10/11 (90.9%) patients were discharged successfully with hospital stays ranging from 4 to 15 days. Neonatal outcomes included one neonatal death, two neonates requiring NICU admission, one intrauterine fetal demise, and one pregnancy terminated due to recurrent PPCM.

## Discussion

PPCM was treated as a diagnosis of exclusion in this study. All women presenting with heart failure during late pregnancy or the postpartum period underwent comprehensive clinical, electrocardiographic, radiographic, and echocardiographic evaluation to exclude alternative causes. Pre-existing cardiomyopathy was ruled out by the absence of prior cardiac disease or symptoms and documentation of new-onset left ventricular systolic dysfunction temporally related to pregnancy or the postpartum period. Transthoracic echocardiography excluded structural, valvular, congenital, ischemic, hypertensive, and pulmonary causes of heart failure, demonstrating global left ventricular systolic dysfunction without significant valvular lesions or regional wall motion abnormalities. Myocarditis and pulmonary embolism were considered unlikely based on clinical presentation, biomarker profiles, and imaging findings. Only women with new-onset left ventricular systolic dysfunction without an identifiable secondary cause were included.

Most patients presented in the antepartum period (54.5%), supporting ESC guidance that PPCM is not restricted to the final month of pregnancy [7]. This is clinically important, as antepartum cases are frequently misdiagnosed as respiratory or hypertensive disorders, leading to delayed referral. Preclampsia is the most common associated risk factor (54.5%), and multiple gestation was present in 22.2% of patients, consistent with established PPCM risk profiles.

The pathophysiology of PPCM is thought to involve pregnancy-related oxidative stress, inflammatory activation, and individual susceptibility. Increased myocardial workload in late pregnancy leads to excess reactive oxygen species production, which in susceptible women promotes cleavage of prolactin into a cardiotoxic 16-kDa fragment [8-11]. This fragment exerts anti-angiogenic, pro-apoptotic, and pro-inflammatory effects, contributing to microvascular dysfunction and impaired myocardial contractility [9]. Experimental data linking prolactin cleavage to endothelial microRNA-146a transfer to cardiomyocytes provide a mechanistic explanation for myocardial dysfunction. Elevated inflammatory markers, including CRP and interleukin-6, reflect systemic inflammation and correlate with disease severity [11,12].

NT-proBNP elevation primarily reflects myocardial wall stress and neurohormonal activation and serves as a marker of disease severity rather than etiology [1,13]. Higher CRP levels were associated with lower LVEF and more severe disease [9]. In one patient with markedly elevated NT-proBNP (29,200 pg/mL) and CRP (>320 mg/L), LVEF improved from 15% to 20% at diagnosis to 51% at six months, highlighting the prognostic value of biomarkers in monitoring severity and recovery.

Emerging evidence supports a genetic predisposition to PPCM, with overlap with dilated cardiomyopathy. Variants in genes related to sarcomeric structure, cytoskeletal integrity, and mitochondrial function suggest pregnancy acts as a “second hit” unmasking latent cardiomyopathy. This underscores the potential role of genetic counseling and testing in selected high-risk patients [14].

Endomyocardial biopsy is not routinely indicated, as no specific histopathological features define PPCM. Therefore, diagnosis remains clinical and echocardiographic, emphasizing careful exclusion of secondary causes [5].

Early recovery was observed in most survivors, with mean LVEF improving from 35.3% at diagnosis to 41.4% at discharge. At six months, 80% achieved complete myocardial recovery (LVEF >50%), while 20% had persistent left ventricular dysfunction even at 18 months. One woman with prior PPCM successfully completed a subsequent pregnancy without relapse after complete recovery, reinforcing evidence that normalized left ventricular function before conception is associated with lower recurrence risk. In contrast, another woman with recurrent PPCM at 21 weeks’ gestation required pregnancy termination and had persistent left ventricular dysfunction, necessitating permanent contraception.

One maternal death (9.1%) occurred due to refractory cardiogenic shock. The patient exhibited multiple ESC-defined high-risk features, including severe left ventricular dysfunction, biventricular involvement, chamber dilatation, pulmonary hypertension, markedly elevated NT-proBNP, delayed referral, and hemodynamic instability, explaining the poor outcome despite aggressive management.

Neonatal outcomes were largely determined by prematurity and maternal instability, with a NICU admission rate of 22.2% and one neonatal death in an extremely preterm infant. Delayed referral and the severity of maternal cardiac dysfunction likely contributed to an adverse neonatal prognosis. Breastfeeding was encouraged, as it is safe in PPCM even with standard heart failure therapy. Postpartum contraceptive counseling emphasized avoidance of estrogen-containing methods, with preference for progestin-only options such as implants or levonorgestrel-releasing intrauterine systems. Women with persistent left ventricular dysfunction were strongly advised against future pregnancy, while those with recovered function were counseled regarding preconception assessment and close multidisciplinary follow-up in line with current ESC guideline recommendations.

This study has several limitations. It was a single-center retrospective study with a small sample size, which limits the generalizability of the findings. Financial constraints may have influenced the prognosis. All laboratory findings were not available for all patients. Despite these limitations, the study provides valuable clinical findings, management, and outcomes in a tertiary care setting.

## Conclusions

PPCM is an important cause of heart failure in pregnancy and the postpartum period, and is often diagnosed late due to the overlap of symptoms with physiological changes of pregnancy. Most patients presented with severe left ventricular dysfunction but showed improvement with timely management. Increased clinical awareness and diagnosis are crucial in improving maternal and neonatal outcomes.
